# Stigmatizing attitudes and causal beliefs for depression and social anxiety among adolescents in Bermuda

**DOI:** 10.1007/s00127-025-02971-8

**Published:** 2025-08-06

**Authors:** Daniel Cavanagh, Laura M. Hart, Shawnee Basden, Nicola Reavley

**Affiliations:** 1 Centre for Mental Health and Community Wellbeing, Melbourne School of Population and Global Health, University of Melbourne, Melbourne, Australia; 2 Department of Arts and Science, Bermuda College, Paget, Bermuda

**Keywords:** Stigmatizing attitudes, Causal beliefs, Depression, Anxiety, Adolescents, Caribbean

## Abstract

**Purpose:**

Stigmatizing attitudes towards individuals with mental illness are common among adolescents. Limited research exists on stigmatizing attitudes and causal beliefs about common mental disorders in adolescent populations in the Caribbean. This study seeks to understand the stigmatizing attitudes and beliefs about the causes of depression and social phobia (social anxiety) among adolescents in Bermuda.

**Methods:**

This cross-sectional study surveyed students aged 10–19 years attending middle or high school in Bermuda. Online surveys conducted between November 2022 and June 2023 gathered data including demographics and symptoms of depression or anxiety, as well as correct problem recognition, stigmatizing attitudes and causal beliefs about a mental health problem described in a vignette.

**Results:**

A total of 2,522 adolescents in Bermuda (70% of eligible participants) provided valid data for the depression (*n* = 1277) or social anxiety (*n* = 1245) vignette. Across both vignettes, reporting female gender was associated with less stigmatizing attitudes, while adolescents who identified their race as Black or Minority reported more stigmatizing attitudes. About three in ten adolescents reported a reluctance to disclose either social anxiety or depression. Respondents were more likely to believe in a psychosocial cause than a chemical imbalance cause of mental illness.

**Conclusion:**

This study suggests there is a need to address stigmatizing attitudes among young people in Bermuda. In particular, anti-stigma campaigns need to be targeted to changing attitudes among those with higher stigma, including males or those reporting Black or Minority race.

## Introduction

Stigmatizing attitudes towards individuals with mental illness are common among adolescents [1–3]. Stigma is a major concern for adolescents with mental illness as it creates a dual burden of coping with their symptoms and the stigma that causes them to face negative stereotypes, prejudice and discrimination [4]. In addition, mental illness stigma is a major barrier to help-seeking [5, 6], especially for adolescents and young people [7]. This is particularly problematic as adolescence is the stage of life where mental disorders often have first onset [8] and help-seeking is low [9, 10]. In turn, delays in help seeking can lead to greater severity and impairment [11] among other unfavourable outcomes [12].

Stigma has been described as an overarching term with three core components: problems of (ignorance), problems of attitudes (prejudice) and problems of behaviour (discrimination) [13]. Several studies have explored stigmatizing attitudes in adolescents and young people [1, 14, 15]. These studies typically examine both personal stigma, which is sometimes termed public stigma, and refers to one’s own perceptions of others and perceived stigma, which refers to one’s beliefs about how others perceive an individual [16]. Personal stigma is of particular concern as higher levels are associated with lower rates of help-seeking [5], with similar findings shown in adolescents [15]. Personal and perceived stigma involve complex attitudes. For example, the Depression Stigma Scale [17] has components relating to attitudes about a person with mental illness as *weak-not-sick* and *dangerous/unpredictable*, while the Social Distance Scale [18] includes items relating to the desire for individuals to distance themselves from those with mental illness.

The desire to reduce stigmatizing attitudes towards people with mental illness has led to a number of national and international awareness campaigns such as *Time to Change* in England or *beyondblue: the national depression* initiative in Australia [19]. Many of these campaigns have emphasised the biochemical causes of mental illness in an effort to challenge the belief that individuals are to blame for their illness [20]. The impact of these campaigns on causal beliefs is important as these beliefs are predictors of treatment preference, engagement and outcomes [21–23]. Causal beliefs vary widely, with some beliefs emphasising psychosocial factors such as a stressful life event, biochemical factors such as a chemical imbalance in the brain, or spiritual factors such as immoral lifestyle. Past research has explored the relationship between causal beliefs and stigmatizing attitudes [24]. Multiple reviews have reported problematic associations between the endorsement of biochemical and biogenetic causes for mental illness and stigmatizing attitudes, such as an increased desire for social distance from affected persons and an increased belief they are dangerous or unpredictable [19, 25].

A small number of studies have explored stigmatizing attitudes among adolescents in the Caribbean. One study in Jamaica found stigmatizing attitudes were reported as a major barrier to help-seeking for these adolescents [26]. The desire for social distance has also been reported in studies with adolescents in the region, irrespective of whether the participants believed in biochemical or psychosocial explanations for mental illness [27]. These findings are supported by qualitative research from Jamaica which found those with mental illness are often described as ‘mad,’ ‘weak’ or ‘dangerous’ and it has been commonly reported that individuals want to avoid those with mental illness [28]. Moreover, while a recent systematic review [29] highlights the impact of stigma on help-seeking in the Caribbean region, none of these studies investigated factors associated with stigmatizing attitudes among adolescents. In studies with adolescents from high income contexts, the impact of demographic characteristics on stigmatizing attitudes has been limited to investigating age and gender [30], with most studies indicating male adolescents hold more stigmatising attitudes than female adolescents [31–33]. However less is known about the impact of race, which may be important as higher stigmatizing attitudes have been found among those who report their race as Black compared to those who report their race as White [34].

Other findings from the study reported in this paper indicated that there is a high prevalence of depression and anxiety symptoms among adolescents in Bermuda [35]. This is concerning as Bermuda’s mental healthcare system was recently reviewed in a situational analysis of small island developing states in the Caribbean [36]. The analysis highlighted the prevalence of stigmatizing attitudes towards people with mental illness. Indeed, previous research reported that stigmatisation of people with mental illness in print media actually increased, not decreased, between 1991 and 2011, in contrast to developed countries such as the UK and USA [37]. The high prevalence of depression and anxiety symptoms among adolescents and the prevalence of stigmatising attitudes in the general population highlight the need to better understand stigmatising attitudes among adolescents in Bermuda. Researching stigmatising attitudes among adolescents allows for an exploration of these developing attitudes that can inform school-based mental health education. As such, this study seeks to understand the prevalence of stigmatizing attitudes and causal beliefs related to depression and anxiety disorders among adolescents in Bermuda, and how they differ according to age, gender, race, the presence of depression and anxiety symptoms and the ability to correctly recognize depression or social anxiety in a vignette.

## Method

### Study population and data collection

This cross-sectional study aimed to survey all adolescent students attending middle or secondary schools in Bermuda. Bermuda is located in the mid-Atlantic Ocean and has a population of approximately 64,000 [38]. According to records provided by schools from the 2022–2023 Academic Year the potential target population was estimated to be 3,593 adolescents aged 10–19 years [K Richards, personal communication, 2 March 2023] [39]. Students were recruited through all but one of the 16 middle (grades M1 through M3) and high (Grades S1 through S4) schools in Bermuda. These schools consisted of seven private, six public (government-funded) and two government-funded ‘alternative education’ and one privately funded ‘alternative education’ schools. The alternative education schools cater to students who are not engaged in mainstream education or experience severe learning disabilities and have complex care needs. One of the government-funded alternative education schools was excluded as students were unable to provide assent. Additional schools were excluded from participation on the basis of very small sample sizes (home schools) or having students outside the eligible age range (community college). A detailed breakdown of the school system in Bermuda, including which schools were surveyed, is provided in [35].

Online surveys were administered during regular class time under teacher supervision between 3 November 2022 and 1 June 2023. The survey involved 22 questions including 7 matrix-style questions that totaled 56 items. The survey took approximately 14 min to complete. Approval for the study was obtained from the University of Melbourne Human Research Ethics Committee (ID: 23177) and the Bermuda Hospitals Board Institutional Review Board. All procedures were performed in compliance with relevant Bermuda laws and school policies. The study used an opt-out parental consent process whereby parents/guardians were considered to have consented to their child participating unless they returned an opt-out form to their child’s school. To ensure parents/guardians were aware of the research, an extensive information campaign was conducted by the research team involving publications in local newspapers and radio interviews at the beginning and in the middle of data collection. Student assent was obtained by having students indicate their willingness to participate on the landing page of the survey. To maintain privacy, a unique Research ID was generated by the research team for each potential participant and schools assigned these to students within their school. Further details about data collection are provided in [35]. An Advisory Board consisting of local clinical professionals was established to provide feedback on the survey, and to ensure culturally safe and relevant practices were put in place during survey administration.

### Measures

The online survey was hosted by the survey platform Qualtrics [40]. It gathered information about students’ demographic characteristics, mental health literacy and mental health first aid beliefs, stigmatizing attitudes, help-seeking intentions and barriers and symptoms of depression and anxiety. The survey presented one of two vignettes that were adapted from the Australian Youth National Survey of Mental Health Literacy and Stigma [31]. The vignettes depicted a fictional peer of the same age, gender and race as the respondent with descriptions of symptoms of either major depression, such as depressed mood and sleep disturbances, or social anxiety, such as fear of social situations and negative evaluations, as per DSM5 and ICD11 [41]. Social anxiety was selected due to its high prevalence compared to generalized anxiety disorder among adolescents [42]. Indeed, globally rates of this social anxiety have been increasing, which has disproportionately affected young people [43]. The vignettes have been previously used in national studies [31], including with adolescents [44]. The Advisory Board recommended the names of the characters in the original vignette be changed from ‘John’ and ‘Jenny’ to ‘Cameron’ and ‘Mikayla’ to be more racially inclusive of the demographic population in Bermuda. Moreover, before administering the survey, diagnostic validity of the vignette was assessed by having four local psychologists identify what they believed to be the problem in the vignette. All psychologists correctly identified the problems described in the vignettes. Within the survey, respondents were randomized to receive either the depression or the social anxiety vignette to understand stigmatising attitudes associated with each of these disorders.

The focus of this paper involves the use of the vignettes to examine respondents’ stigmatizing attitudes and causal beliefs, as well as how these outcomes are associated with age, gender, race and the presence of moderate to severe symptoms of depression or anxiety. A copy of the depression vignette of ‘Cameron’ and the social anxiety vignette of ‘Mikayla’ are provided in Supplementary Table 1.

### Dependent variables

To assess stigmatizing attitudes, respondents were provided with a set of statements on personal attitudes towards a peer described in a vignette. These statements were taken from the Australian Youth National Survey of Mental Health Literacy and Stigma involving young people aged 15 to 25 years [31], and were originally adapted from the Depression Stigma Scale [17]. The statements included: (1) ‘they could snap out of it if they wanted,’ (2) ‘their problem is a sign of personal weakness,’ (3) ‘their problem is not a real medical illness,’ (4) ‘they are dangerous,’ (5) ‘it is best to avoid them so that you don’t develop this problem yourself,’ (6) ‘their problem makes them unpredictable,’ (7) ‘I would not tell anyone if I had a problem like them.’ Ratings of each statement were made on a 5-point Likert scale ranging from ‘strongly agree’ (5) to ‘strongly disagree’ (1).

Previous factor analyses using data from the Australian Youth National Survey of Mental Health Literacy and Stigma have shown that the stigma scale has two components: *weak-not-sick* (items (1), (2), (3) and (5) above) and *dangerous/unpredictable* (items (4), (5) and (6) above), and that the item ‘I would not tell anyone if I had a problem like them’ did not load on any scale [20, 45]. Scale scores were calculated for each of the scales by summing each of the scale items ratings, while the individual item was scored separately as *reluctance to disclose*. The Pearson correlation between the scales was *r* = 0.58 (*p* < 0.01).

Respondents reported their willingness to have contact with the peer described in the vignette using an adapted Social Distance Scale from the Australian Youth National Survey of Mental Health Literacy and Stigma involving young people aged 15 to 25 years [31]. The items rates respondents’ willingness to (1) ‘to go out with them on the weekend,’ (2) ‘to work on a project with them,’ (3) ‘to invite them around to your house,’ (4) ‘to go to their house,’ (5) ‘to develop a close friendship with them.’ This scale was developed and validated prior to the mass adoption of social media among adolescents globally [46]. As such, to modernize the scale an additional item was included (6) ‘to connect with them on social media.’ Qualitative feedback from pilot testing indicated that adolescents had no issues of comprehension related to the addition of this item. Each statement was rated on a 5-point Likert scale ranging from (1) ‘yes, definitely,’ (2) ‘yes, probably,’ (3) ‘not sure’, (4) ‘probably not,’ to (5) ‘definitely not.’ A scale score was calculated by summing the ratings. The internal consistency scores are provided for the *weak-not-sick* (Cronbach alpha = 0.694) and *dangerous/unpredictable* (Cronbach alpha = 0.643) scales. Pearson correlations between the social distance scale and the stigma scales were as follows: *weak-not-sick* *r* = 0.246 (*p* < 0.001) and *dangerous/unpredictable* *r* = 0.289 (*p* < 0.001). As the social media item was added to the Social Distance Scale, answers to the social distance items were subjected to principal components factor analysis without rotation. Inspection of eigenvalues and the scree plot were used to determine the number of components to retain. Principal components factor analysis yielded one factor with an eigenvalue > 1. This factor explained 61.% of the variance. The Cronbach alpha was 0.87.

To assess causal beliefs, respondents were asked about two possible causes of the problem described in the vignette. They were asked whether they agreed that (1) ‘their [peer’s] problem was caused by a stressful life event’ (psychosocial) and (2) ‘their [peer’s] problem was caused by a chemical imbalance in the brain.’ These two causal beliefs were selected to be consistent with previous research in the region [27], with a particular interest in biochemical explanations due to the problematic nature of public health campaigns related to this explanation [19]. Ratings of each statement were made on a 5-point Likert scale ranging from ‘strongly agree’ (5) to ‘strongly disagree’ (1). Responses were then dichotomised to contrast respondents who ‘strongly agreed’ or ‘agreed’ (1), to other respondents (0).

### Independent variables

Demographic variables were first assessed, including participants’ age (month and year of birth), gender (*Male*,* Female* or *identifying with another term)* and race *(Black*,* White*,* Portuguese*,* Mixed*,* Asian or Pacific Islander*, or *Other*).

Participants were then provided with one of the vignettes. To assess the ability to correctly recognize the problem in the vignette, respondents were asked *what*,* if anything*,* do you think is wrong with [Cameron/Mikayla]?* For the depression vignette, those who wrote ‘depressed’ ‘depression’ or ‘depressive’ were considered to have correctly recognised the disorder and assigned a score of 1. For the social anxiety vignette, those who wrote ‘social anxiety’, ‘social anxiety’, ‘anxiety’, ‘anxious’, ‘anxiety attacks’ or ‘anxiety disorder’ were considered to have correctly identified the disorder and assigned a score of 1. Those who did not correctly recognise the problem were assigned a score of 0. This coding frame was taken from the Teen Mental Health First Aid studies [47, 48].

To assess symptoms of depression and anxiety among respondents the PHQ-9 (Patient Health Questionnaire-9) and the General Anxiety Disorder – 7 (GAD-7) [49, 50] were used. These are validated screening tools that assess symptoms over the past two weeks according to the *Diagnostic and Statistical Manual of Mental Disorders 5th edition* [41] criteria. Respondents reporting ‘moderate,’ ‘moderately severe’ or ‘severe’ symptoms of either depression or anxiety – indicated as having a score of 10 or above on either scale - were coded as having these symptoms present and assigned a score of 1, while those who scored below 10 were assigned a score of 0. Following feedback from the Advisory Board, the item on suicidality was removed from the PHQ-9. Previous research notes that the omission of this item does not lead to any reductions in the validity or reliability of the instrument [51]. For the PHQ-8, a cut-point score of 10 and above has a sensitivity of 100% and specificity of 95% for major depressive disorder [52]. The GAD-7 sensitivity and specificity using a cut score of 10 are 89% and 82%, respectively [50].

### Statistical analyses

Demographic characteristics were first summarized across both vignettes as either mean ± standard deviation for age, or n (%) for all other variables. For gender, 1.7% (*N* = 39) of respondents reported ‘I identify with another term’ (19 younger adolescents, 20 older adolescents, 3 missing). For the purposes of analyses, respondents who identified their race as Mixed (17.3%), Portuguese (6.9%), Asian or Pacific Islander (2.0%) or Other (2.9%) were recoded into a category labelled ‘Minority.’ Of 2,716 students who assented to the survey, the 2,522 respondents who provided valid data for sociodemographic, stigmatising attitudes and causal belief items were randomly assigned to either the depression vignette (*n* = 1277) or the social anxiety vignette (*n* = 1245).

Binary logistic regressions were conducted to examine whether the independent variables of age, gender, race, correct problem recognition and the presence of moderate to severe depression/anxiety symptoms were associated with causal beliefs in response to the depression vignette, and again separately for the social anxiety vignette. With the exception of age, which was a continuous variable, all independent variables were categorical. The independent variables were simultaneously entered into the model (*Italics* indicate reference category): gender (*male*, female) and race (*White*, Black, Minority), correct problem recognition (*incorrect*, correct) moderate to severe depression or anxiety symptoms (*absence*, presence). In addition, a chi-squared test was used to reveal differences in the proportion of adolescents who endorsed the chemical imbalance belief compared to the psychosocial belief for each vignette.

Finally, linear regression analyses were conducted to examine the relationship between the independent variables described above and causal beliefs with the *weak-not-sick* scale, the *dangerous/unpredictable scale*, the *reluctance to disclose* item and the Social Distance Scale. The reference category for both belief that the problem was caused by a stressful life event and a chemical imbalance in the brain was *other* (referring to those that did not agree or strongly agree).

Statistical analyses were conducted in SPSS version 29. For regression analyses, only data from respondents who assented to the survey and completed all items related to stigmatizing attitudes, causal beliefs, demographic characteristics, correct problem recognition and depression and anxiety symptoms were included in the analysis. Respondents who assented but did not complete all items were excluded from analyses (*N* = 95).

## Results

### Demographic characteristics and causal beliefs

A total of 2,522 adolescents in Bermuda (ages ranging 10–19 years) assented and provided valid data. As shown in Table 1, the majority of respondents identified as Black, female and attended a private school. Almost half of the respondents (48%) agreed with the psychosocial cause for the problem described in the depression vignette as opposed to 13% who agreed with the chemical imbalance explanation. For the social anxiety vignette, less than one third (30%) agreed with the psychosocial cause as opposed to 9% who agreed with the chemical imbalance explanation. A chi-squared test revealed significantly more respondents agreed with the psychosocial cause than the chemical imbalance cause in the social anxiety vignette (*p* < 0.001).

**Table 1:** Demographic and other characteristics of the sample

Variable	All participants (*N* = 2522)	Depression vignette (*n* = 1277)	Social anxiety vignette (*n* = 1245)
Age, y (mean ± SD)	13.8 ± 2.0	13.8 ± 1.9	13.8 ± 2.0
Gender, *n* (%)			
Female	1308 (51.9)	660 (51.7)	648 (52.0)
Male	1175 (46.6)	598 (46.8)	577 (46.3)
I identify with another term	39 (1.7)	19 (1.5)	20 (1.6)
Race, *n* (%)			
Black	1109 (44.0)	577 (45.2)	532 (42.7)
White	675 (26.8)	329 (25.8)	346 (27.8)
Minority*	734 (29.1)	370 (29.0)	364 (29.2)
School, *n* (%)			
Private	1504 (59.6)	753 (59.0)	751 (60.3)
Government	1018 (40.4)	524 (41.0)	494 (39.7)
Ability to correctly recognize disorder in vignette, *n* (%)			
Correct problem recognition	1158 (45.9)	722 (56.5)	642 (51.6)
Moderate to severe depression/anxiety symptoms, *n* (%)			
Present symptoms	870 (34.5)	445 (34.8)	425 (34.1)
Absent symptoms	1540 (61.1)	776 (60.8)	764 (61.4)
Causal belief of problem described in vignette, *n* (%)			
Stressful life event	998 (39.6)	618 (48.4)	380 (30.5)
Chemical imbalance in the brain	270 (10.7)	163 (12.8)	107 (8.6)

*Minority accounted for those who identified as ‘Mixed’ (*n* = 438), ‘Portuguese’ (*n* = 176), ‘Asian or Pacific Islander’ (*n* = 52) or ‘Other’ (*n* = 68)

### Stigmatizing attitudes

**Fig. 1:**
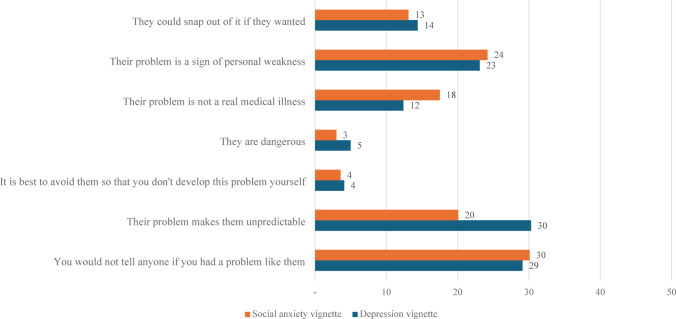
Percentage (%) of respondents who ‘agree’ or ‘strongly agree’ with items from the stigma scale, in response to vignettes depicting an adolescent peer with either depression or social anxiety

**Fig. 2:**
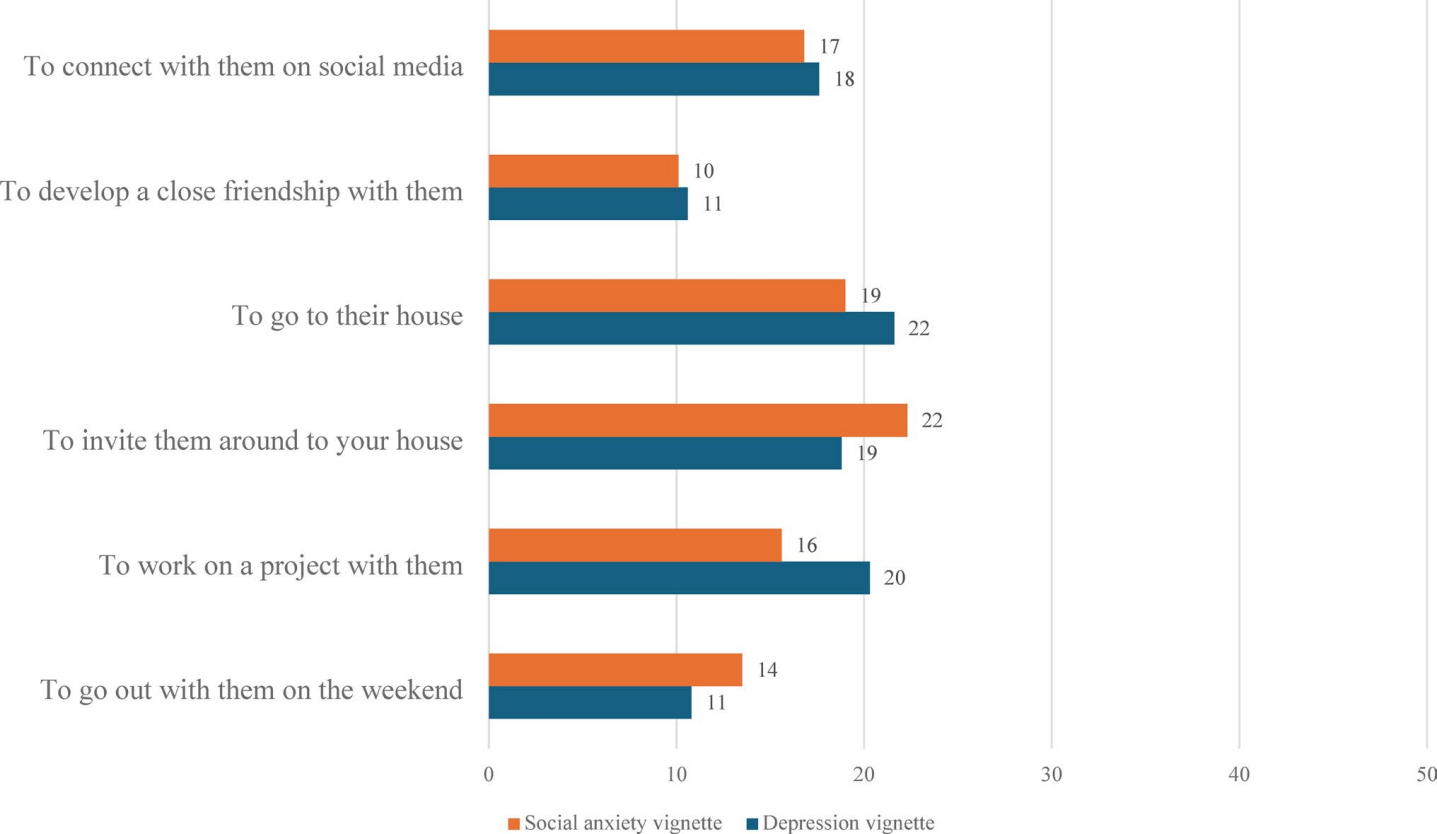
Percentage (%) of respondents desiring various types of social distance from the person described in the vignette

For the depression vignette, the most commonly endorsed item from the stigma scales was ‘their problem makes them unpredictable’ while the least commonly endorsed was ‘it is best to avoid them so that you don’t develop this problem yourself.’ In the social anxiety vignette, the most commonly endorsed stigmatized belief was ‘you would not tell anyone if you had a problem like them’ while the least commonly endorsed was ‘they are dangerous.’ These results provided in Figs. 1 and 2.

### Variables associated with stigmatizing attitudes

For the depression vignette, older age, female gender, correct problem recognition and the presence of moderate to severe depression/anxiety symptoms were all associated with lower scores on the *weak-not-sick* scale. Black and Minority adolescents scored higher on this scale compared to White adolescents, as did those who agreed with the belief that the problem was caused by a stressful life event. Female gender and the presence of moderate to severe depression/anxiety symptoms were associated with lower scores on the *dangerous/unpredictable* scale. Black and Minority adolescents scored higher on this scale compared to White adolescents, as did those who agreed that the problem was caused by a chemical imbalance. Older age and the presence of moderate to severe depression/anxiety symptoms were both associated with higher scores on the *reluctance to disclose* item. Finally, female gender, correct problem recognition, the presence of moderate to severe depression/anxiety symptoms and agreement of the chemical imbalance belief were all associated with lower scores on the *social distance* scale.

For the social anxiety vignette, female gender, correct problem recognition and the presence of moderate to severe depression or anxiety symptoms were all associated with lower scores on the *weak-not-sick* scale. Black and Minority adolescents scored higher on this scale compared to White adolescents, as did those who agreed the problem was caused by a stressful life event. Female gender and correct problem recognition were associated with lower scores on the *dangerous/unpredictable* scale. Adolescents who identified as Black scored higher on this scale compared to White adolescents, as did those who agreed the problem was caused by a stressful life event or a chemical imbalance in the brain. The presence of moderate to severe depression/anxiety symptoms was associated with higher scores on *reluctance to disclose* item. Finally, female gender, the presence of moderate to severe depression or anxiety symptoms and agreeing with the chemical imbalance belief were all associated with lower scores on the *social distance* scale. The detailed results are provided in Table 2.

**Table 2:** Summary of linear regressions (-coefficients) exploring differences in stigmatizing attitudes according to associated variables for the depression and social anxiety vignettes

Variable	Depression vignette (*N* = 1167)				Social anxiety vignette (*N* = 1144)			
	Weak-not-sick	Dangerous/unpredictable	Reluctance to disclose	Social distance	Weak-not-sick	Dangerous/unpredictable	Reluctance to disclose	Social distance
Older age (in years)	−0.13**	−0.01	0.08*	−0.04	−0.05	−0.04	0.03	0.05
Female (reference group: Male)	−0.28**	−0.25**	0.01	−0.16**	−0.22**	−0.21**	0.01	−0.15**
Identified with another term (reference group: Male)	−0.11**	−0.08*	0.09*	−0.03	−0.06	−0.06	0.02	−0.09*
Black (reference group: White)	0.23**	0.18**	0.00	−0.05	0.22**	0.15**	0.00	0.08*
Minority (reference group: White)	0.19**	0.08	0.07	−0.01	0.15**	0.09	−0.01	0.04
Correct problem recognition (reference group: incorrect)	−0.19**	−0.03	−0.02	−0.07	−0.31**	−0.13**	0.03	−0.11**
Presence depression/anxiety symptoms (reference group: absence)	−0.12**	−0.07	0.20**	−0.11**	−0.08*	−0.01	0.19**	−0.07
Agreement that problem is caused by stressful life event (reference group: did not agree)	0.06	0.01	0.02	−0.03	0.08*	0.11**	0.07	0.02
Agreement that problem caused by chemical imbalance in the brain (reference group: did not agree)	0.003	0.09*	0.05	−0.05	0.06	0.15**	−0.02	−0.01

Participants were randomized to receive either the depression or social anxiety vignette

Presence depression/anxiety symptoms as indicated by those scored ≥ 10 on the PHQ-8 or the GAD-7

* *p* < 0.01

** *p* < 0.001

### Variables associated with causal beliefs

All independent variables were significantly associated with psychosocial or chemical imbalance causal beliefs. Regarding psychosocial causal beliefs, respondents who reported moderate to severe symptoms of depression/anxiety, older age, female gender and correct problem recognition were less likely to agree than disagree, in comparison to their counterparts, that depression was caused by a stressful life event. Students who identified as Black or of Minority race were more likely to agree than disagree, as compared to those who identified as White, that depression was caused by a stressful life event. In the social anxiety vignette, female respondents were less likely to agree than disagree that this problem was caused by a stressful life event compared to males. Those reporting moderate to severe depression/anxiety symptoms were more likely to agree than disagree that social anxiety was caused by a stressful life event compared to those not reporting these symptoms.

Regarding the chemical imbalance causal belief, for both the depression and social anxiety vignettes, older age and those who reported moderate to severe depression/anxiety symptoms were more likely to believe in this causal belief compared to their counterparts. Black adolescents were less likely than White adolescents to agree than disagree that depression was caused by a chemical imbalance, whereas female adolescents were less likely to agree than disagree with this causal belief compared to males. The detailed results are shown in Table 3.

**Table 3:** Summary of logistic regressions for variables associated with causal statements about a peer with depression or social phobia as depicted in a vignette

Variable	Stressful life event						Chemical imbalance in the brain					
	Depression vignette (*N* = 1177)			Social anxiety vignette (*N* = 1154)			Depression vignette (*N* = 1177)			Social anxiety vignette (*N* = 1154)		
	OR	99% CI		OR	99% CI		OR	99% CI		OR	99% CI	
Older age (in years)	0.91*	0.84	0.99	0.99	0.91	1.08	1.16*	1.03	1.30	1.24**	1.08	1.42
Female (reference group: Male)	0.63**	0.46	0.86	0.73	0.51	1.04	0.79	0.49	1.27	0.57	0.31	1.03
Identified with another term (reference group: Male)	0.32	0.08	1.31	1.49	0.41	5.45	0.48	0.07	3.54	0.33	0.02	5.06
Black (reference group: White)	1.64**	1.12	2.40	0.94	0.63	1.41	0.56*	0.32	0.98	0.77	0.40	1.48
Minority (reference group: White)	1.68**	1.11	2.54	0.94	0.61	1.45	0.84	0.48	1.49	0.72	0.35	1.47
Correct problem recognition (reference group: incorrect)	0.68*	0.49	0.93	0.85	0.60	1.21	1.16	0.71	1.88	1.40	0.76	2.57
Presence depression/anxiety symptoms (reference group: absence)	1.03	0.74	1.44	1.39	0.97	1.99	1.74*	1.08	2.81	1.58	0.88	2.83

Participants were randomised to receive either the depression or social anxiety vignette

Logistic regression tested the proportion of students who responded ‘strongly agree’ or ‘agree’ to the causal statement vs. all other responses: OR (99% CI)

Presence depression/anxiety symptoms as indicated by those scored ≥ 10 on the PHQ-8 or the GAD-7

* *p* < 0.01

** *p* < 0.001

## Discussion

Our study found stigmatizing attitudes were common among a large minority of adolescents in Bermuda, and that they are more prevalent among males and those who report their race as Black or of a Minority. Promisingly, those who could correctly recognize the problem in the vignette were less likely to hold stigmatizing attitudes, however, those who reported moderate to severe depression/anxiety were the most likely to report a reluctance to disclose. We also found that a higher percentage of adolescents endorsed a psychosocial explanation for depression and social anxiety than a chemical imbalance explanation.

While methodological differences make comparisons difficult, our results suggest that personal stigmatising attitudes may be less common among adolescents in Bermuda than they are in other contexts [1, 53]. In the current study, less than one in three adolescents reported stigmatizing attitudes for either vignette. Moreover, less than one in five adolescents reported stigma and desire for social distance on most items. Similar to studies in Australia, for the social anxiety vignette, scores were higher on the *weak-not-sick* subscale and lower on the *dangerous/unpredictable sub*scale [54]. While it is promising to see lower than expected prevalence of stigmatizing attitudes, it is a matter of concern that about three in ten adolescents report reluctance to disclose either social anxiety or depression. The reluctance to disclose mental health problems is consistent with previous research in the region in small island contexts [36]. Small populations make disclosure particularly difficult due to issues of confidentiality, which has been highlighted as a major barrier to high-quality mental healthcare in Bermuda [55]. As such, while personal stigmatising attitudes may not be as common as in other contexts, it is plausible that perceived stigmatising attitudes would be even more prevalent in these contexts. Moreover, in this study, adolescents who reported depression/anxiety symptoms were less likely to believe the character in the vignette was weak-not-sick. As such, the high prevalence of depression and anxiety symptoms reported among adolescents in Bermuda may explain lower personal stigmatising attitudes than in other contexts [1, 35, 53].

In terms of gender and racial differences, our results support findings from a systematic review that indicated male adolescents hold more stigmatizing attitudes than females [32], including a greater desire for social distance [56]. Similarly, we found that males had more stigmatizing attitudes than those who did not identify as male or female. Our results also support the findings that non-White adolescents were more likely to hold stigmatizing attitudes [32, 56]. Interestingly, one study in the USA found that Black boys reported more stigmatizing attitudes than non-Latino boys and white girls, while non-Latina Black girls and boys and Latina girls and boys wanted more social separation from peers with mental illness than Non-Latino white girls [57]. Our finding that Black adolescents wanted more social distance to the person described in the social anxiety vignette supports these findings.

Our finding that adolescents in Bermuda were more likely to endorse a psychosocial explanation than a chemical imbalance explanation is unsurprising. Despite many campaigns globally emphasizing chemical imbalance as causes of mental illness, adolescent populations are more likely to believe in psychosocial causes of mental illness [58]. Multiple reviews have reported problematic associations between the endorsement of biochemical causes for mental illness and stigmatizing attitudes, particularly those relating to dangerousness [19, 25]. As we also found that endorsement of a chemical imbalance was associated with more stigmatizing attitudes, it may be encouraging that only very few adolescents endorsed this belief. Moreover, it’s likely that as adolescents age, they are exposed to the concept of a chemical imbalance as a cause of mental illness, leading to the greater endorsement of this belief among older adolescents. This appears to be the case among those who reported moderate to severe depression/anxiety symptoms as they were the most likely to endorse the chemical imbalance belief for depression. Interestingly, we found that endorsement of psychosocial causes increased the belief that the person in the social anxiety vignette was *dangerous/unpredictable.* As both beliefs were associated with more stigmatizing attitudes, further research would be useful in exploring the relationships between causal beliefs and different dimensions of stigmatizing attitudes among adolescents. Limited research is available to explain why females were less likely to endorse a psychosocial explanation of depression or why this belief was more commonly endorsed among those who identified as Black or of a Minority race. It is possible that these sociodemographic groups are more likely to endorse other causal beliefs such as spiritual explanations, although further research would be needed to explore this. In any case, as causal beliefs are predictors of treatment preference, engagement and outcomes [22, 23, 59], it would be beneficial for future research to investigate what is driving differences in causal beliefs among adolescents in Bermuda.

### Implications

Our findings suggest efforts to reduce stigma need to be targeted to changing attitudes among those with higher stigma, including males and those reporting Black or of a Minority race. Moreover, any campaigns should avoid exacerbating problematic trends in mental health promotion efforts [60], for example avoiding the promotion of the ‘brain disease’ explanation for depression [61]. The association of correct problem recognition with reduced stigma points to the importance of evidence-based programs that improve the ability to recognize the symptoms of disorders, such as teen Mental Health First Aid (tMHFA) [47, 62, 63]. The presence of moderate to severe depression/anxiety symptoms was most strongly associated with the reluctance to disclose. It is problematic that the adolescents most in need of support were associated with reporting the greatest reluctance to seek help. As such, future research should investigate the help-seeking intentions and barriers to care among adolescents experiencing these symptoms.

### Strengths and limitations

This study is the first to investigate mental illness stigma among adolescents in Bermuda. A strength of this study is the sample size in that 70% of all eligible adolescents, those attending middle and high schools, across the nation provided valid data. Other findings from this study in Bermuda found that depression and anxiety symptoms are highly prevalent in adolescents [35]. Therefore, the findings from this study are useful in understanding barriers to help-seeking, and what components of stigma anti-stigma campaigns should target. A limitation of this study relates to the use of Western diagnostic criteria to describe the symptoms of depression and social anxiety, which may not acknowledge cultural and racial differences in the manifestation of symptoms. For example, studies have found differences in the presenting of symptoms of depression [64–66], including that those who identify as Black present more somatic symptoms, such as sleep disturbance, compare to those who identify as White [67]. Similarly, this study only included the use of one item in each case to investigate psychosocial and chemical imbalance causal beliefs. Neither focused on spiritual causes which are common in populations in the Caribbean [68], and have been linked to higher depression stigma [69]. Further research using a broader set of causal beliefs [70, 71] would be useful in better understanding determinants of culturally relevant causal beliefs, and how they influence stigmatizing attitudes and the desire for social distance. Finally, the use of a vignette to determine stigmatising attitudes decreases ecological validity [72]; in real life individuals likely get more information about an individual and their symptoms and this may affect their attitudes.

### Conclusion

Stigmatising attitudes among adolescents in Bermuda are relatively common, particularly among males and those who report their race as Black or of a Minority. Adolescents in Bermuda are more likely to endorse a psychosocial explanation for depression and social anxiety than a chemical imbalance explanation. The reluctance to disclose depression or social anxiety is concerning in relation to future help-seeking among these adolescents. This study highlights the need to address stigma among adolescents in Bermuda, in particular through anti-stigma campaigns tailored to the local context. Our findings suggest psychoeducation interventions should target groups of young people with higher stigma, including males and those who identify as Black or of a Minority race. Further research would be useful to understand help-seeking intentions and barriers among adolescents, particularly those reporting mental illness.

## Data Availability

Access to the data described within the manuscript and supplementary files can be granted upon an email request to the authors.
